# Novel canonical and non-canonical viral antigens extend current targets for immunotherapy of HPV-driven cervical cancer

**DOI:** 10.1016/j.isci.2023.106101

**Published:** 2023-02-02

**Authors:** Xu Peng, Isaac Woodhouse, Gemma Hancock, Robert Parker, Kristina Marx, Julius Müller, Silvia Salatino, Thomas Partridge, Annalisa Nicastri, Hanqing Liao, Gary Kruppa, Karin Hellner, Lucy Dorrell, Nicola Ternette

**Affiliations:** 1 Centre for Immuno-Oncology, Nuffield Department of Medicine, University of Oxford, OX3 7DQ Oxford, UK; 2 Nuffield Department of Medicine, University of Oxford, OX3 7FZ Oxford, UK; 3 The Jenner Institute, University of Oxford, OX3 7DQ Oxford, UK; 4 Bruker Daltonics, Fahrenheitstraße 4, 28359 Bremen, Germany; 5 Wellcome Centre Human Genetics, University of Oxford, OX3 7BN Oxford, UK; 6 Nuffield Department of Women’s and Reproductive Health, University of Oxford, John Radcliffe Hospital, OX3 9DU Oxford, UK; 7 Immunocore Ltd., OX14 4RY Abingdon, UK

**Keywords:** Immunology, Cell biology, Cancer

## Abstract

Current immunotherapeutic approaches for human papillomavirus (HPV)-driven cervical cancer target the viral oncogenes E6 and E7. We report viral canonical and alternative reading frame (ARF)-derived sequences presented on cervical tumor cells, including antigens encoded by the conserved viral gene E1. We confirm immunogenicity of the identified viral peptides in HPV-positive women, and women with cervical intraepithelial neoplasia. We observe consistent transcription of the E1, E6, and E7 genes in 10 primary cervical tumor resections from the four most common high-risk HPV subtypes (HPV16, 18, 31, and 45), suggesting the suitability of E1 as therapeutic target. We finally confirm HLA presentation of canonical peptides derived from E6 and E7, and ARF-derived viral peptides from a reverse-strand transcript spanning the HPV E1 and E2 genes in primary human cervical tumor tissue. Our results extend currently known viral immunotherapeutic targets in cervical cancer and highlight E1 as an important cervical cancer antigen.

## Highlights

• Peptides derived from HPV-E1 are presented by cervical tumor cells
• Out-of-frame translation events are a source for viral antigen presentation
• E1-derived viral antigens are immunogenic in HPV-positive patients
• Viral epitopes are sourced from non-canonical viral transcription and translation events

## Graphical abstract

**Figure:**
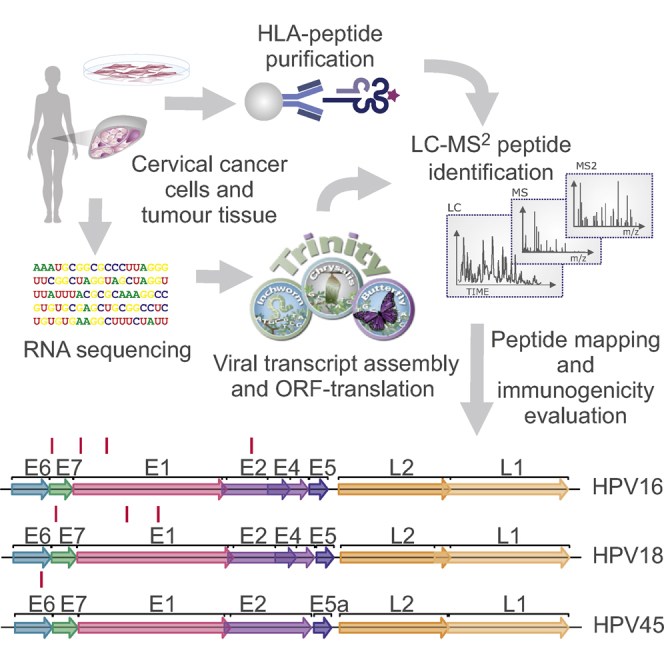

## Introduction

Human papillomaviruses (HPVs) are small DNA viruses, of which there are more than 170 types, including 16 types that are designated “high risk” or oncogenic.^1^^,^^2^ High-risk types are present in more than 99% of cervical cancers, which is the fourth most common type of female cancer worldwide.^3^^,^^4^^,^^5^^,^^6^ Among the types of high-risk HPV, HPV16, HPV18, HPV45, and HPV31 are the most common, with 59%, 12%, 4.8%, and 3.7% prevalence, respectively.^7^ Over 600,000 women were diagnosed with cervical cancer, and 341,831 deaths were recorded worldwide in 2020.^3^

HPVs rely on the host cell replication proteins to mediate viral DNA and protein synthesis. The small HPV genome, approximately 8 kb in size, can be divided into six early genes (*E1*, *E2*, *E4*, *E5*, *E6*, and *E7*), which have regulatory function and play a role during the viral life cycle, and two late genes (*L1* and *L2*) that form the viral capsid.^8^ Most HPV infections are subclinical and are cleared by the human immune system within 2 years of acquisition; however, persistent infection with HPV can lead to cell transformation and accumulation of mutations over time, which can ultimately induce the development of a cancerous lesion.^9^

The HPV oncogenic proteins E6 and E7 are crucial in the induction and maintenance of cellular transformation and are expressed in most HPV-associated cervical cancers. High-risk E6 proteins prevent cell growth inhibition by targeting the tumor suppressor p53 for degradation via the ubiquitin pathway,^10^^,^^11^ which prevents apoptosis and extends cell life. Similarly, the HPV E7 protein binds to retinoblastoma protein family members and targets them for degradation, which drives the E2F-dependent induction of the cell life cycle and induces S phase and cell proliferation.^11^^,^^12^^,^^13^ Both E6 and E7 can interact with c-Myc and support cell longevity through telomerase reverse transcriptase promotor activation.^14^^,^^15^ As a result, among other interactions, these oncoproteins perturb the control of cell cycle progression, leading to increased cellular proliferation and survival, which contributes to the development and maintenance of HPV-associated cancers.

T cell immunity is important for control of HPV-driven cancers as shown by the increased prevalence of HPV-associated tumors in immunocompromised individuals.^16^ It has further been shown that tumor-infiltrating lymphocytes correlate with improved outcomes in patients with cervical cancer.^17^^,^^18^^,^^19^ Since it is established that HPV E6/E7 are expressed in HPV-driven malignancies to maintain continuous cell survival and proliferation, several therapeutic approaches have been developed and evaluated targeting these viral oncoproteins.

Adoptive T cell therapy selected for E6 and E7 antigens has been shown to be able to induce complete tumor regression in selected treated patients,^20^ and adoptive T cell therapy showed a dominant T cell clonotype targeting HLA-A^∗^02:01-restricted E6_29–36_ (TIHDIILECV) in a patient with metastatic anal cancer, who had been disease free 22 months after successful treatment.^21^ Furthermore, treatment using T cell receptor (TCR)-engineered T cells targeting E7 for patients with metastatic HPV-associated epithelial cancers showed a number of partial responders and robust tumor regression in 6 of 12 patients including 4 of 8 patients with anti-PD-1 refractory disease.^22^

DNA vaccines encoding both E6/E7 fusion genes are more potent than those containing E7 alone in the TC-1 cell line.^23^ In addition, therapeutic vaccination targeting CD40 with HPV E6 and E7 showed protection against TC-1 tumors in human CD40 transgenic mice, and a significant increase of HPV16-specific CD8^+^ CTLs in the tumors.^24^ Results of three proof-of-concept T cell vaccines currently in clinical trials for the treatment of cervical intraepithelial neoplasia (CIN) stage 2 or 3 have been published to date.^25^^,^^26^^,^^27^ Inovio’s DNA vaccine (VGX-3100)^25^ is a mixture of two DNA plasmids encoding optimized HPV16/18 E6/E7 consensus sequences. GX-188E (Genexine Inc.) is a DNA vaccine consisting of a tissue plasminogen activator signal sequence, an Fms-like tyrosine kinase 3 (Flt3)-ligand, and an HPV16/18 E6/E7 fusion antigen.^26^^,^^28^ The Tipapkinogen sovacivec (TS) vaccine is a modified vaccinia Ankara vectored vaccine encoding human cytokine interleukin (IL)-2, and non-oncogenic forms of HPV16 E6 and E7 proteins.^27^ However, the observed clinical benefits are modest, possibly reflecting poor immunogenicity of the vaccine regimens and limited antigenic breadth of the immunogens.

It has been shown in HPV-driven squamous cell carcinoma that immune recognition of other viral antigens is highly relevant. Previous studies have demonstrated high mRNA levels and high titers of serum antibodies against E1, E2, E4, and E5, in addition to E6 and E7 in HPV-driven head and neck cancer.^29^^,^^30^^,^^31^ Two recent studies indicate that HPV proteins other than E6 and E7 are relevant in T cell immune recognition of HPV-positive oropharyngeal cancer (OPC) and head and neck squamous cell carcinoma (HNSCC). Bhatt et al. profiled both CD4^+^ and CD8^+^ T cells that recognize E1, E2, E4, E5, and L1 proteins in patients with OPC, with E1 responses present in most of the patients interrogated in the study. Eberhardt et al. showed presence of HPV-specific T cells in HNSCC, and defined specificity to E2, E5, and E6 in HNSCC.^32^

Since E1-specific T cell responses have been recently shown to be present in patients with cervical cancer,^33^ we here set out to perform an unbiased analysis of the HPV antigenic landscape in cervical cancer tumor cell lines using immunopeptidomics to understand which viral proteins are processed and presented on the tumor surface to T cells.

Our results expand the known landscape of HPV-derived antigens presented on cervical tumor cells to cryptic, out-of-frame translated viral antigens and next to the known viral antigens E6 and E7, extending the source of viral antigens to the genes encoding E1 and E2.

## Results

### Immunopeptidomic analysis yields highly specific HLA-associated peptide data for a panel of five cervical tumor cell lines

We selected a panel of five human cervical cancer cell lines as the basis of our study to investigate the T cell antigen landscape of cervical cancer. CaSki,^34^ DoTc2-4510 (henceforth called DoTc2), and SiHa^35^ cells are HPV16-positive, whereas HeLa^36^ cells are transformed with HPV18. C33^37^ was included as an HPV-negative cervical tumor control cell line. We first measured surface expression of HLA-I and HLA-DR by flow cytometric analysis in all cell lines using pan-HLA class I and class II HLA-DR-specific antibodies. Although all cell lines were positive for HLA class I, only DoTc2 showed additional expression of surface HLA-DR receptors (Figure S1). To confirm the HLA type of each cell line, we performed conventional 4-digit HLA genotyping (Data S1). The cell line panel overall comprises 4 different HLA-A alleles, six HLA-B alleles, and five HLA-C alleles.

**Figure 1:**
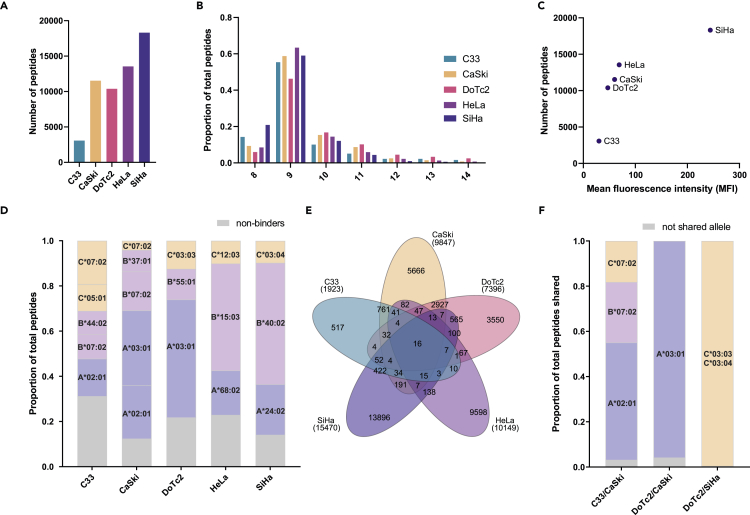
Identification of tumor-associated antigens and neoantigens in cervical tumor cell lines (A) Number of peptide sequences identified. (B) Length distributions of identified peptides in proportion of total identifications. (C) Relationship between the overall number of peptides identified and MFI ratio as determined by flow cytometry. (D) Proportion of peptides predicted to bind to the corresponding HLA present in each line (8–14mer sequences). (E) Overlap of peptide sequences between all cell lines (8–14mer sequences). (F) Proportion of binders to the corresponding common HLA allele(s) of all shared peptides identified in C33 and CaSki, DoTC2 and CaSki, and DoTc2 and SiHa cell lines.

Having confirmed the HLA genotypes and expression levels of HLA across the cell lines, we performed immunopeptidomics experiments for all five cell lines in duplicates. Overall, we identified a total of 56,948 peptides from 10,919 proteins in these experiments, with numbers ranging from 3,072 to 18,306 peptides per sample (median 11,534; Figure 1A). More than 94% of the peptides had the typical length of HLA-I peptides (8–14 amino acids), with the majority of peptides (58%) being 9 amino acids long (Figure 1B). We observed that the higher the HLA-I surface expression levels as measured by flow cytometry (median fluorescence intensity [MFI]), the higher the number of peptides identified in the experiment (Figures 1C and S1). Although the linear correlation between the MFI and peptide number was strong (R^2^ = 0.65), it did not reach statistical significance (p = 0.1).

We also employed advanced neural-network-based algorithms (NetMHCpan v4.1)^38^^,^^39^ to predict HLA binding of the identified peptide sequences. A range of 69%–88% of all peptides detected were predicted to bind to the HLA-A, -B, and -C alleles present in the respective cell lines (median 78%; Figure 1D). The obtained peptide sequences showed a low degree of overlap between the different cell lines, with more than 85% of the identified peptides being unique to one of the five cell lines, which is expected for samples with distinct HLA haplotypes (Figure 1E). We observed a high degree of overlap of sequences for cells expressing the same HLA allele, such as DoTc2 and CaSki cells (HLA-A^∗^03:01, 2,927 peptides in common), C33 and CaSki cells (HLA-A^∗^02:01, B^∗^07:02, C^∗^07:02, 761 peptides in common), and SiHa and DoTc2 (HLA-C^∗^03:03 and HLA-C^∗^03:04, 565 peptides in common). Shared peptides were almost exclusively predicted to originate from one of the alleles that the cell lines had in common (average 97%, Figure 1F).

### RNA sequencing confirms consistent transcription of E1 and E2 in addition to E6 and E7 in the HPV-positive cell lines

**Figure 2:**
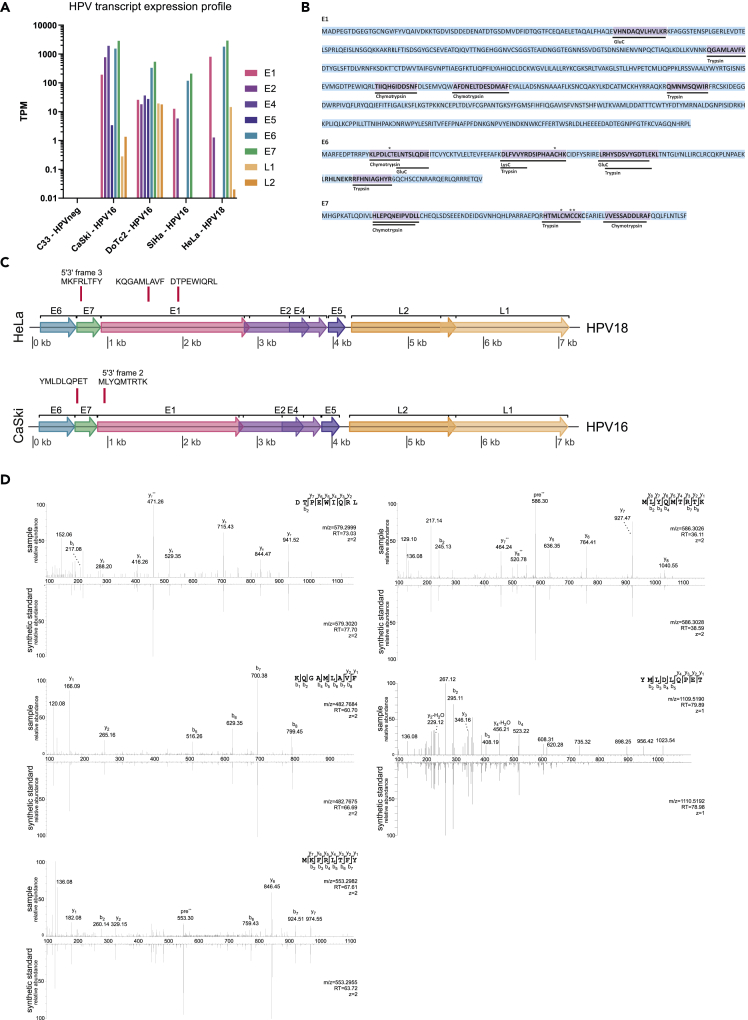
Viral gene expression profile and canonical and alternative reading frame HLA peptide presentation in HPV-transformed cervical cancer cells (A) RNA transcript levels in indicated cell lines. Displayed are read counts per gene for HPV16 (C33, CaSki, DoTc2, SiHa) and HPV18 transcripts (HeLa). (B) Evidence for HPV E1, E6, and E7 protein expression in HeLa cells in the deep proteome dataset PXD004452. Underlined sequences highlighted in violet were identified in the sample for which the indicated enzyme was used for initial protein digestion. Stars depict carbamidomethylation on cysteine residues. (C) Relative position of viral HLA peptides identified mapping to HPV16 and HPV18 viral genomes, respectively. The horizontal line indicates the nucleotide position in the regarding viral genome; horizontal boxes in rainbow colors show the open reading frames for the main viral proteins. Red vertical lines indicate the coding sequence for the identified HLA-associated peptide sequences. (D) LC-MS fragment spectra are shown as measured in the experiment (sample), and measured from the equivalent synthetic peptide sequence counterpart (synthetic standard). The measured mass of the precursor peptide ion ([M+2H]++), charge state (z), and the retention time (RT) at which the peptide ion was selected for fragmentation are stated within each spectral panel. C-terminal fragment ions are indicated as y, N-terminal fragments are designated b.

To confirm HPV status and establish expression levels of HPV-derived transcripts, we performed RNA sequencing across the cell line panel. All HPV16 genes with conventionally established expression (E1, E2, E4, E5, E6, E7, L1, and L2) were detected at the transcriptional level in DoTc2 and CaSki, whereas only transcripts from HPV16 E1, E2, E6, and E7 were detected in SiHa (Figure 2A). Expression of HPV18 transcripts were detected exclusively in HeLa cells (E1, E2, E6, E7, and L1; Figure 2A), as expected. We observed evidence for transcription of E1 and E2 across all cell lines except in C33, which was negative for HPV transcripts as expected (Figure 2A).

### HPV18 E1 protein is expressed in addition to E6 and E7 in HeLa cells

The HeLa cell line is a commonly used proteomics standard for the interrogation of human proteome sequencing depth,^40^ and, as such, there are many deep proteomics datasets available in the public domain. A dataset by the Olsen laboratory (PXD004452),^41^ in which several enzymes were used for digestion to increase sequence coverage and depth, was selected and re-interrogated to validate expression of HPV proteins in HeLa cells. Our analysis confirmed protein expression of HPV18 E1, E6, and E7 proteins in HeLa cells, which had not been reported in the original publication (Figure 2B), in line with the mRNA expression levels characterized in our RNA sequencing datasets.

### E1 and E7 canonical and alternative reading frame (ARF)-derived viral peptide antigens are presented by HLA in cervical cancer cells

To refine our search for HPV-derived HLA peptides, we reconstructed viral transcript sequences by *de novo* assembly of RNA reads using Trinity.^42^ Following a first pass of mapping to the human reference genome, unmapped reads are used as input to Trinity for assembly. After filtration of the output transcript sequences by searching for partial overlaps with the sample-matched HPV genome using BLASTn, we performed six-frame translation of assembled transcripts and concatenated the resulting protein sequences over 8 amino acids in length to the SwissProt reviewed human reference proteome. Using this approach, we identified five peptides from the assembled viral transcripts that mapped to the HPV genome (Figure 2C, Table 1). In HeLa cells we observed three peptide antigens originating from HPV18. KQGAMLAVF (KF9, HLA-B^∗^15:03) and DTPEWQRL (DL8, HLA-A^∗^68:02) were derived from E1, whereas a peptide matching an alternative reading frame (ARF) in E7 was also identified (MKFRLTFY, MY8, HLA-B^∗^15:03, Figure 2C, Table 1). In CaSki cells, we identified two HPV16-associated peptides. YMLDLQPET (YT9), a well-known and characterized T cell antigen^43^ that is predicted to bind to HLA-A^∗^02:01 and MLYQMTRTK (MK9, HLA-A^∗^03:01), which maps to a short ARF within the E1 gene region (Figure 2C, Table 1).

**Table 1:** Tumor-specific antigens derived from viral transcripts identified in cervical cancer cells

HPV Peptide	Rank	HLA allele	−10lgP	HPV protein	Genome ORF position
**HeLa HPV18**					

KQGAMLAVF	0.157	B^∗^15:03	20.48	E1	1,541–1,567
DTPEWIQRL	0.112	A^∗^68:02	21.96	E1	1,937–1,963
MKFRLTFY	0.610	B^∗^15:03	27.8	E7 ARF	654–677

**CaSki HPV16**					

MLYQMTRTK	0.026	A^∗^03:01	34.24	E1 ARF	956–982
YMLDLQPET	0.127	A^∗^02:01	5.11^a^	E7	592–618

ORF, open reading frame.

Rank score: HLA binding score as predicted by NetMHCpan4.1; −10lgP score: peak score indicating the likelihood of correct spectral sequence assignment.

a Below score cutoff set but validated by spectral matching.

### Identified HPV peptides are recognized by T cells from women with persistent HPV infection or cervical intraepithelial neoplasia

**Figure 3:**
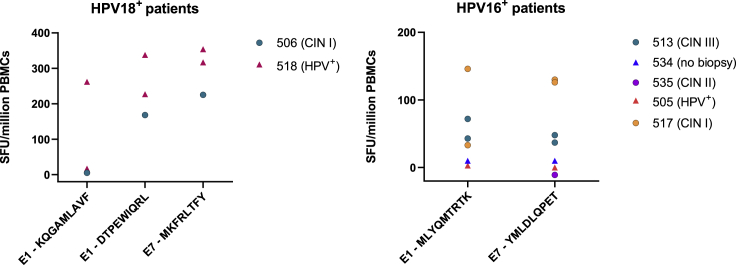
Confirmation of immunogenicity of identified viral and cryptic peptide sequences in HPV-positive women with or without CIN (stages I–III) T cell responses measured by cultured IFN-γ ELISpot to peptides identified in HPV16- and HPV18-positive patients with or without CIN (stages I–III) as indicated in the legend. Mock-stimulated values have been subtracted. Data shown are the mean of 3–5 replicates.

To validate the identified sequences derived from viral transcripts for their potential to elicit immune responses in infected individuals, we generated short-term HPV-specific T cell lines (STCLs) from PBMC from seven women with CIN (grades 1–3) or colposcopic evidence of HPV changes without CIN (five HPV16-positive, and two HPV18-positive on vaginal sampling for HPV DNA). These STCLs were then tested for recognition of the cognate peptide sequences using cultured interferon (IFN)-γ ELISpot assays. We detected a T cell response to each peptide sequence in at least 1 of the 7 patients, confirming that the mass spectrometry (MS)-identified peptides were immunogenic in the context of natural infection (Figure 3). As a negative control, T cells from all seven women were simultaneously expanded using a pool of irrelevant peptides (FEC peptide pool sourced from influenza, Epstein-Barr virus, and cytomegalovirus) and then tested for recognition of the HPV peptide sequences in parallel. No responses were detected.

### HPV E1 is consistently expressed in primary cervical tumor tissue

**Figure 4:**
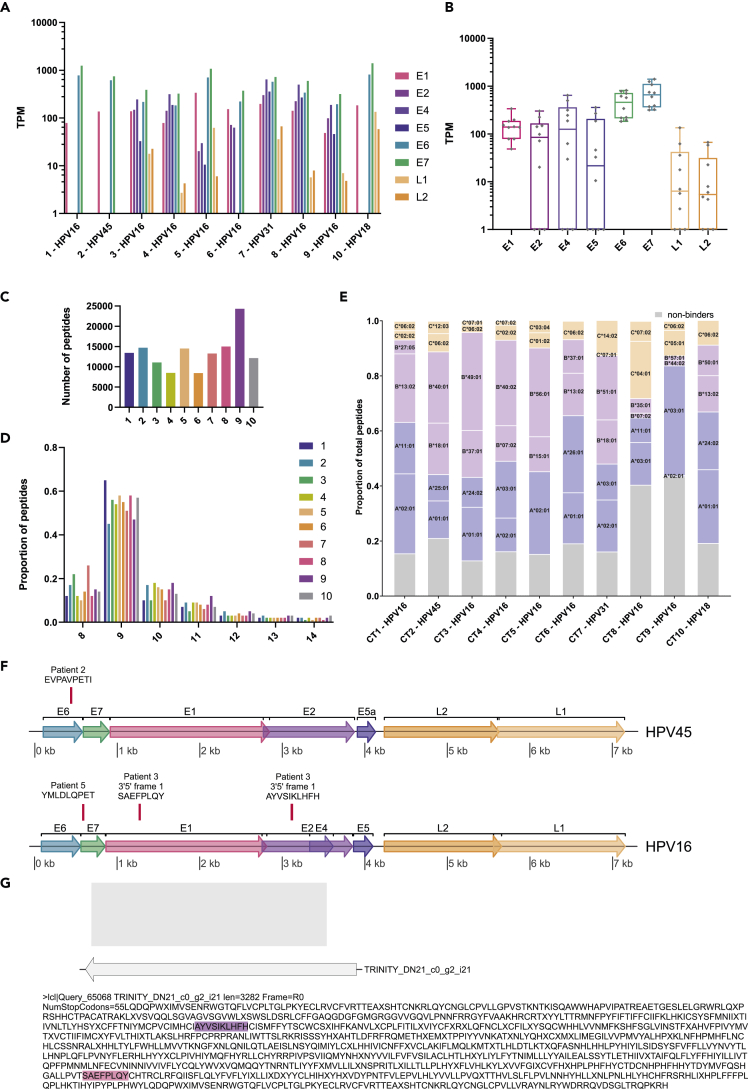
Viral gene expression and canonical and non-canonical viral antigen presentation in primary cervical tumor tissue (A and B) Bar chart (A) and boxplot (B) of transcript copies of the main viral genes across 10 primary cervical tumor samples. Whiskers in boxplot indicate minimal and maximal detected TPM for each viral gene. (C) Number of HLA-associated peptide sequences identified for each tissue. (D) Length distributions of identified peptides in percent of total identifications. (E) Proportion of peptides predicted to bind to the corresponding HLA present in each line (8–14mer sequences). (F) Relative position of viral HLA peptides identified mapping to the HPV16 and HPV45 genome. The horizontal line indicates the nucleotide position in the regarding viral genome; horizontal boxes in rainbow colors show the open reading frames for the main viral proteins. Red vertical lines indicate the location of the coding sequence for the identified HLA-associated peptide sequences. (G) Relative position of the Trinity transcript (gray arrow) encoding the non-canonical peptides derived from the E1 and E2 gene regions. The gray box indicates the relative position to the HPV16 genome in Figure 4F and the encoded reading frame 1 is shown in single amino acid code. The identified HLA-associated peptide sequences are highlighted in the color representing the viral gene region of their origin.

To confirm the clinical relevance of our finding that HPV E1 can be presented on cervical tumor cells, we performed RNA sequencing on 10 primary tumor tissue samples. We first determined the HPV genotype by mapping viral RNA reads to reference genomes reported in PAVE.^2^ Reads were mapped to HPV16 in 7 tumor samples, and to HPV18, 31, and 45 in one sample each (Figure 4A). We then analyzed the relative transcript expression levels for all HPV viral genes and found that only E1, E6, and E7 were consistently transcribed across all 10 primary tissue samples (Figures 4A and 4B), whereas other early proteins and the structural proteins had more varying expression across the analyzed tissue cohort. L1 and L2 expression was lowest across the tissues, whereas E5 expression was only present in 6 of 10 tumor tissues, rendering it the least consistently expressed viral protein in this study. These data confirm the high relevance of E1 as a potential viral antigen target in primary HPV tumors, as it is highly expressed in cancer tissue from all four most frequent high-risk HPV subtypes, HPV 16, 18, 31, and 45.

### HPV E1 and E2 genes are sources for non-canonical HLA-presented antigens in human cervical tumor tissue

Finally, we performed immunopeptidomic profiling on the obtained cervical tumor tissues. We obtained between 8,439 and 24,298 peptides with a length of 8–14 amino acids per tissue (Figures 4C and 4D), and between 56% and 86% of peptides were predicted to bind to the regarding HLA alleles present in each patient (Figure 4D, patient HLA types listed in Data S2). We again performed Trinity *de novo* assembly to reconstruct the patients’ individual viral transcript profile and to characterize the potential viral sequence pool. Within those peptide sequences that were predicted to be derived from HLA presentation (Figure 4E), we sequenced four peptides originating from the assembled viral transcripts (Table 2). We were able to sequence a novel canonical peptide derived from HPV45-E6, EVPAVPETI, which was predicted to bind to HLA-A^∗^25:01 (patient 3, Table 2). We also again observed the known HLA-A^∗^02:01 epitope derived from HPV16-E7, YMLDLQPET (patient 5) (Table 2, Figure 4F). We further observed two additional peptides originating from E1 (SAEFPLQY, HLA-A^∗^01:01) and E2 (AYVSIKLHFH, HLA-A^∗^24:02) viral genes in patient 3, both peptides derived from a reverse-strand transcript spanning the E1 and E2 gene region (Figures 4F and 4G, Table 2).

**Table 2:** Tumor-specific antigens derived from viral transcripts identified in cervical cancer tissue

HPV peptide	Rank	HLA allele	−10lgP	HPV protein	Genome ORF position
**Patient 2, HPV45**					

EVPAVPETI	0.237	A^∗^25:01	25.91	E6	410–336

**Patient 3, HPV16**					

SAEFPLQY	0.336	A^∗^01:01	25.35	E1 ARF	1,293–1,270
AYVSIKLHFH	0.718	A^∗^24:02	21.85	E2 ARF	3,132–3,103

**Patient 5, HPV16**					

YMLDLQPET	0.127	A^∗^02:01	17.24^a^	E7	592–618

Rank: HLA binding rank score as predicted by NetMHCpan4.1; −10lgP score: peaks score indicating the likelihood of correct spectral sequence assignment.

a Below 5% FDR score cutoff set, but validated by spectral matching.

These data confirm the relevance of non-canonical viral antigens in cervical cancer, and underline the importance of their consideration in the development of therapeutic approaches to cervical cancer.

## Discussion

The first HLA class I-restricted HPV T cell epitopes published in the literature were identified through T cell immunogenicity screening in HLA-A^∗^02:01-positive healthy donors of predicted peptide sequences from E6 and E7.^44^ The list was expanded by B^∗^18:01-bound sequences from E6 and E7,^45^ and several HLA class I-bound E7 peptides were mapped in CaSki cells using a targeted liquid chromatography (LC)-MS^3^ approach by screening a library of HPV16 E7-derived peptides that were predicted to bind to HLA-A2.^46^^,^^47^ Such targeted MS approaches are highly sensitive but are limited by the need to establish a list of peptide candidates through prediction, and the need to synthesize these molecules for inclusion in the synthetic peptide library required for MS acquisition. The characterized E7_11–19_ (YMLDLQPET) in the latter study, which was also detected in our study, was later shown to be present in primary cervical cancer biopsies using the same targeted MS approach.^48^

It was reported previously that cervical cancer cell lines exhibited antigen-processing defects for the E6_29-38_ (TIHDIILECV) peptide; however, peptide-specific CD8^+^ T cells were able to kill CaSki indicating presentation of this peptide in CaSki cells.^49^ This peptide was not identified in our study, likely due to the presence of a cysteine residue in the peptide. Cysteine is a highly reactive amino acid that has a high likelihood of undergoing modifications during sample preparation. It is well-documented that cysteine-containing peptides are often underrepresented in immunopeptidomic studies.^50^^,^^51^^,^^52^

While profiling viral RNA expression levels in our cervical cell line panel, we detected expression of all main HPV proteins in DoTc2 cells, in contrast to earlier reports and current literature that suggest the cells to be HPV-negative.^53^^,^^54^ We would like to suggest here to carefully re-test the cell line if used as an experimental model, and to regard the biological safety level as unknown until a full characterization is completed.

With our unbiased proteogenomics discovery approach combining next-generation RNA sequencing and immunopeptidomics, we report for the first time the direct discovery of HPV16 and HPV18-derived peptides presented by HeLa and CaSki cells. While confirming presentation of the known HLA-A^∗^02:01 peptide YMLDLQPET derived from HPV16 E7 in CaSki cells, we further identified a peptide derived from an HPV16 ARF in the E1 gene transcript (MLYQMTRTK). In HeLa cells, we also detected two peptides derived from HPV18 E1, KQGAMLAVF and DTPEWIQRL, predicted to bind to B^∗^15:03 and A^∗^68:02, respectively, together with a peptide derived from an alternative reading frame of E7 (MKFRLTFY), which was mapped to B^∗^15:03. It is to be noted that A^∗^68:02 is an HLA-A2 supertype, suggesting that the ARF peptide could be presented by A2-positive patients. We also confirmed the presence of HPV18 E1 protein in deep proteome data of HeLa cells, as previously shown by western blot.^55^

These results demonstrate the importance of the E1 gene as a source for antigens presented in cervical cancer beyond E6 and E7. We confirmed that peptides are immunogenic in HPV-positive women, demonstrating the relevance of E1 T cell recognition in HPV infection and early disease. The observed HPV-specific T cell responses in PBMC from these individuals are consistent with their known HPV exposure and are thus likely to be mediated by effector and or memory T cells rather than naive T cells that have undergone differentiation *in vitro*. The generation of antigen-specific T cells from naïve precursors by *in vitro* priming typically requires stimulation by antigen-loaded mature dendritic cells, followed by *in vitro* expansion for a longer culture period than that employed here. These findings are also in close alignment with a study that investigated the frequency of E1-specific responses in peripheral blood in patients with cervical squamous cell carcinoma.^33^

E1 is highly conserved across papillomavirus subtypes, making it an attractive therapeutic target. The protein is the only viral protein with a defined enzymatic activity and exhibits an ATP-dependent helicase function that recruits components of the cellular replication machinery for viral genome replication.^56^^,^^57^ E1 can also directly downregulate the expression of interferons (IFNs), which are key mediators of the cellular antiviral immune response, and further promote the expression of IFN-stimulated genes that encode molecules with a multitude of antiviral effector functions.^58^^,^^59^

To confirm the clinical relevance of E1 peptide presentation in primary human cervical tumors, we proceeded to profile RNA expression of viral transcripts and confirmed that E1 transcription is maintained in 10 of 10 primary human cervical tumor resections, including all four most frequent high-risk HPV subtypes (HPV16, HPV18, HPV31, and HPV45). Other early proteins were less consistently expressed across the analyzed cohort, suggesting a central role of E1 in HPV-driven tumors, and highlighting E1 as the most suitable additional viral protein target in addition to E6 and E7.

Finally, the relevance of ARF-derived viral peptides was confirmed in primary cervical tissue with the identification of two peptides derived from a reverse-sense transcript spanning the E1 and E2 gene region, suggesting that non-canonical transcription and translation events contribute to cervical tumor antigenicity in patients.

Together, we conclude that non-canonical antigens derived from HPV, most importantly from the viral gene E1, are important additional targets to be considered for future immunotherapeutic approaches to cervical cancer.

### Limitations of the study

LC-MS^2^ remains a discovery technology, and the actual extent of viral antigens presented on cervical cancer cells and tissue remains to be determined. Although our study extends the known targetable viral antigen space to the viral gene E1, and further highlights the presentation of viral antigens from ARFs and non-canonical antisense transcripts, the mechanism and contribution of such non-canonical viral transcription and translation events to viral antigen presentation in HPV-transformed cells and their suitability as targets for immunotherapy has yet to be fully characterized.

## STAR★Methods

### Key resources table

**Table:** 

REAGENT or RESOURCE	SOURCE	IDENTIFIER
**Chemicals, peptides, and recombinant proteins**		

KQGAMLAVF	Genscript	HPV-18-E1_KF9
DTPEWIQRL	Genscript	HPV-18-E1_DL9
MKFRLTFY	Genscript	HPV-18-E7_MY8
MLYQMTRTK	Genscript	HPV-16-E1_MK9
YMLDLQPET	Genscript	HPV-16-E7_YT9

**Deposited data**		

Cell line immunopeptidomics data	ProteomeXchange Consortium via the PRIDE	https://doi.org/10.6019/PXD028738

**Experimental models: Cell lines**		

Anti-HLA class I (clone W6/32)	ATCC	ATCC Cat#: HB-95; RRID:CVCL_7872
Anti-HLA-DR (clone L243)	ATCC	ATCC Cat#: HB-55; RRID:CVCL_4533
CaSki	ATCC	ATCC Cat#: CRL-1550; RRID:CVCL_1100
DoTc2-4510	ATCC	ATCC Cat#: CRL-7920; RRID:CVCL_1181
SiHa	ATCC	ATCC Cat#: HTB-35; RRID:CVCL_0032
C-33-A	ATCC	ATCC Cat#: HTB-31; RRID:CVCL_1094
HeLa	ATCC	ATCC Cat#: CCL-2; RRID:CVCL_0030

**Software and algorithms**		

Peaks	Bioinformatics Solutions	X Pro
Trim_Galore	http://www.bioinformatics.babraham.ac.uk/	v0.6.2
FastQC	http://www.bioinformatics.babraham.ac.uk/	v0.11.9
STAR	Dobin et al., 2013^60^	v2.7.3a
Samtools	Li et al., 2009^61^	v1.10
GATK	McKenna et al., 2010^62^	v4.1.7.0
Subread	Liao et al., 2014^63^	v1.6.4
HPV-EM	Inkman et al., 2020^64^	v1.0
Trinity	Grabherr et al., 2011^43^	v2.11.0
seqinr	Guy et al., 2010^65^	v1.0
BLASTn	NCBI	version 2020

### Resource availability

#### Lead contact

Further information and requests for resources and reagents should be directed to and will be fulfilled by the Lead Contact, Nicola Ternette, nicola.ternette@ndm.ox.ac.uk.

#### Materials availability

This study did not generate new unique reagents.

### Experimental model and subject details

#### Human PBMC and tissue samples

PBMC were obtained from women aged 25–55 years with HPV16 or HPV18 infection confirmed by genotyping of viral DNA in a self-taken vaginal sample, with or without colposcopic abnormalities associated with HPV infection. These women were participants in an observational study of natural immune responses to high-risk HPV, which was approved by the South West – Central Bristol Research Ethics Committee (ref. 16/SW/0331) and has been described previously.^66^ All women gave written informed consent. Cervical tumor tissue samples were obtained from Tissue Solutions Ltd. from women with grade 2–3 tumors aged 24–59 years old. Ethical approval was granted by the Central University Research Ethics Committee (CUREC) of the University of Oxford under reference R68126/RE001.

#### Cervical cancer cell lines and culture

C33-A (carcinoma, HTB-31, ATCC) and SiHa (squamous cell carcinoma, HTB-35, ATCC) were cultured in Minimum Essential Medium (Sigma) supplemented with 10% heat-inactivated fetal bovine serum (FBS), 2 mM L-glutamine, 1 mM pyruvate, 0.1 mM Non-Essential Amino Acids Solution and 100U penicillin/ml. HeLa (adenocarcinoma, CCL-2, ATCC) and DoTc2-4510 (carcinoma, CRL-7920, ATCC) were cultured in Dulbecco’s Modified Eagle’s Medium (Sigma) supplemented with 10% heat-inactivated FBS, 2 mM L-glutamine and 100U penicillin/ml. CaSki (squamous cell carcinoma, CRL-1550, ATCC) were cultured in RPMI 1640 (Sigma) supplemented with 10% heat-inactivated FBS, 2 mM L-glutamine, 1 mM pyruvate, 0.1 mM HEPES Solution and 100U penicillin/ml. All lines were grown at 37°C in 5% CO_2_.

### Method details

#### Quantification and statistical analysis

For mass spectrometric analysis, spectral sequence annotation was controlled using a decoy database search build-in with the Peaks software (Bioinformatics Solutions). The probability scores of each spectral annotation was calculated using a linear discriminative function (LDF), which was converted into a P-value that represents the probability of the annotation to be a non-random assignment. For example, a given score cut-off of −10log_10_(P) = 15 is equivalent to a p value of p = 0.032, and 10log_10_(P) = 20 is equivalent to a p value of p = 0.01. Please see more details in the section “LC-MS2 data analysis”.

#### Flow cytometry

Cells were washed and stained with antibodies specific for pan-HLA-I and HLA-DR (W6/32, L243, Biolegend) for 20 min at room temperature. Cells were then washed and fixed in 4% PFA. Staining data were acquired on an LSRFortessa and analysis was performed on FlowJo software version 10.6.2.

#### MHC class I immunoprecipitation

1 mL Protein A-Sepharose beads (GE Healthcare) were washed in 50 mM borate, 50 mM KCl (pH 8.0) solution and then incubated with 2 mg of pan-HLA-I antibody (W6/32) slowly rotating for 1 h at 4°C. The beads were washed in a column format with 0.2 M triethanolamine (pH 8.2), and the bound antibody was cross-linked by incubation with 40 mM dimethyl pimelimidate dihydrochloride (DMP) (Sigma) (pH 8.3) for 1 h at room temperature. Ice-cold 0.2 M Tris buffer (pH 8.0) was added to the mixture to stop the reaction. Unbound antibody was washed off the column by washing with 0.1 M citrate (pH 3.0), and the column was equilibrated in 50 mM Tris (pH 8.0) for further use. 5 × 10^8^ cells pellets were lysed by using 10 mL lysis buffer (0.5% IGEPAL 630, 150 mM NaCl, 50 mM Tris, pH 8.0, supplemented with protease inhibitor cocktail (Roche)), and mixed for 30 min. The lysate was centrifuged at 300 g for 10 min to remove nuclei and then at 15,000 g for 60 min to pellet other insoluble material. 1 mL W6/32 cross-linked to protein A-Sepharose beads (GE) was added to cleared lysates for 1h, and beads were washed with 50 mM Tris buffer (pH 8.0) containing first 150 mM NaCl, then 450 mM NaCl, and next no salt. HLA-peptide complexes were eluted by using 5 mL 10% acetic acid and dried.

#### High-performance liquid chromatography

After immunoprecipitation, peptide-HLA complexes were resuspended in 120 μL loading buffer (0.1% trifluoroacetic acid (TFA), 1% acetonitrile (ACN) in water). Samples were fractioned by reverse-phase (RP) high-performance liquid chromatography (HPLC) using an Ultimate 300 HPLC system (Thermo Scientific) supplemented with a 4.6- by 50-mm Pro-Swift RP-1S column (Thermo Scientific). Samples were loaded onto the column and eluted using a 10 min linear gradient from 3% to 30% ACN in 0.1% TFA at a flow rate of 500 nL/min, and elution was monitored by light absorbance at 280 nm. Fractions were collected in 1-min intervals. Alternate fractions were combined in two final fractions (odd and even), dried, resuspended in 20 μl of loading buffer and analyzed by LC-tandem mass spectrometry (LC-MS^2^).

#### Liquid chromatography mass spectrometry (LC- MS^2^)

Orbitrap Fusion Lumos Tribrid Mass Spectrometer (Thermo Scientific): Peptides were analyzed by LC-MS^2^ using an Ultimate 3000 RSLCnano System supplemented with a PepMap C18 column, 2 μm particle size, 75 μm × 50 cm (Thermo Scientific) directly interphased with an Orbitrap Fusion Lumos Tribrid mass spectrometer (Thermo Scientific). A 60 min linear gradient from 3% to 25% ACN in 5% DMSO/0.1% formic acid at a flow rate of 250 nL/min was applied for peptides elution. Peptide ions were introduced to the mass spectrometer using an Easy-Spray Source at 2000 V. Detection was performed with a resolution of 120,000 for full MS (300–1500 *m*/*z* scan range), and precursors were selected using TopSpeed ion selection within a 2 s cycle time, and an quadrupole isolation width of 1.2 amu for fragmentation. For MS^2^ acquisition the resolution set at 30,000 and high-energy collisional dissociation (HCD) energy was set at 28 for peptides with 2-4 charges and 32 for peptides that were singly-charged.

TimsTOF SCP – (Bruker Daltonics): Cervical primary tumor samples were analyzed by liquid chromatography (nanoElute, Bruker Daltonics) online coupled to a timsTOF SCP - Bruker Daltonics. One microliter of each sample was loaded directly onto the separation column (Aurora, 25 cm × 75 μm, 1.7 μm, C18 column (IonOpticks, Australia) using 0.1% formic acid at a loading pressure of 800 bar for 9 min at 50°C. Separation of peptides was performed at 50°C using a linear gradient of 2–20% ACN in 0.1% acetic acid over 60 min and 20–37% in additional 6 min at a flow rate of 150 nL/min. Electrospray ionization conditions with the CaptiveSpray source (Bruker Daltonics) were 180°C, 1400 V capillary voltage and 3 L/min dry gas. Mass spectrometric analysis was performed in a data-dependent (DDA) PASEF mode. One MS survey TIMS-MS and 10 PASEF MS/MS scans were acquired as per acquisition cycle. Ion accumulation and ramp time in the dual TIMS analyzer was set to 166 ms each. Peptides were analyzed in an ion mobility range from 1/K0 = 1.7 Vs cm2 to 0.7 Vs cm-2 and *m*/*z* range of 100–1,700. Multiply charged ions and singly charged ions with *m*/*z* ≥ 700 with an intensity were included for precursor selection. Precursors with a minimum threshold of 500 arbitrary units (a.u.) were fragmented and re-sequenced until reaching a “target value” of 20,000 a.u. Optimized collision energies for immunopeptidomics analysis were used: 70 eV at 1/K0 = 1.7 Vs cm^−2^; 40 eV at 1/K0 = 1.34 Vs cm^−2^; 40 eV at 1/K0 = 1.1 VS Vs cm^−2^; 30 eV at 1/K0 = 1.06 Vs cm^−2^; 20 eV at 1/K0 = 0.7 Vs cm^−2^.

#### LC-MS^2^ data analysis

LC- MS^2^ datasets were analyzed using Peaks v10.6. For in-house datasets the following parameters were set: Orbitrap Fusion Lumos Tribrid Mass Spectrometer (Thermo Scientific): Precursor mass tolerance: 5 ppm; fragment mass tolerance: 0.05 Da; digestion: none; fixed and variable modifications: none.

TimsTOF SCP – (Bruker Daltonics): Precursor mass tolerance: 20 ppm; fragment mass tolerance: 0.05 Da; digestion: none; fixed and variable modifications: none. 6-frame translations of Trinity assemblies mapping to the HPV genome were concatenated with the reviewed human entries in Uniprot (Swissprot version April 2018, containing 20,328 entries). For cell line data, a threshold of −10lgP = 15 was applied across all datasets, and the FDR was consistently reported as <5% (C33: 4.83%; Caski: 1.79%; Dotc2: 1.81%; Hela: 1.22%; Siha: 0.87%). For primary tumor data, score thresholds were chosen to achieve a global FDR of 5% (CT1: −10lgP = 18.9; CT2: −10lgP = 18.1; CT3: −10lgP = 20.4; CT4: −10lgP = 20.7; CT5: −10lgP = 18.7; CT6: −10lgP = 21.6; CT7: −10lgP = 18.8; CT8: −10lgP = 19.0; CT9: −10lgP = 17.4; CT10: −10lgP = 20.6).

For PXD004452 data analysis the following parameters were selected: precursor mass tolerance: 20 ppm; fragment mass tolerance: 0.05 Da; digestion: specific to the indicated enzyme used for digestion; fixed modifications: carbamidomethylation; variable modifications: none.

#### DNA purification and exome sequencing

Genomic DNA was extracted cell lines using DNeasy Blood & Tissue Kits (Qiagen). DNA extraction was performed following the manufacturer’s instructions from 5 × 10^6^ cells each. DNA concentration and purity were measured by Nanodrop (NanoDrop 2000 spectrophotometer, Thermo Fisher). Exome sequencing of all samples was performed in the Wellcome Center Human Genetics, Oxford Genomics Center on an Illumina HiSeq 4000 system using the SureSelect kit v6 (Agilent).

#### RNA purification methods

Total RNA was extracted from the five cervical cell lines using RNeasy Mini Kits (Qiagen). RNA extraction was performed following the manufacturer’s instructions from 5 × 10^6^ cervical cancer cells RNA concentration and purity were measured by Nanodrop (NanoDrop 2000 spectrophotometer, Thermo Fisher). RNA sequencing of all samples was performed in the Wellcome Center Human Genetics, Oxford Genomics Center on an Illumina HiSeq 4000 system using the SmartSeq2 workflow.

#### HPV typing and RNA *de novo* viral transcript assembly

Raw RNA sequencing reads had adaptors removed and low-quality bases trimmed using Trim_Galore v0.6.2 (http://www.bioinformatics.babraham.ac.uk/projects/trim_galore) and quality control metrics assessed using FastQC v0.11.9 (http://www.bioinformatics.babraham.ac.uk/projects/fastqc). A custom STAR (v2.7.3a)^67^ genome reference was created utilising UCSC hg38 genome sequence together with relevant HPV genome sequences from PaVE (https://pave.niaid.nih.gov/).^2^ Reads were then mapped to this custom genome reference using STAR with two passes, sorted and indexed by Samtools v1.10.^60^ Duplicates were marked using GATK v4.1.7.0 ^61^ MarkDuplicates function and viral-mapped reads were quantified using featureCounts from Subread v1.6.4 ^62^ to perform HPV-typing. This was then confirmed using the HPV genotyping tool HPV-EM.^63^ Read counts mapping to HPV genes are a direct output of HPV-EM and are displayed without transformation.

Unmapped reads from STAR were pooled with viral-mapped reads and used as inputs for Trinity v2.11.0^43^ [10] for *de novo* transcript assembly. These resulting transcripts were filtered by aligning to each sample’s respective HPV genome using NCBI BLASTn.^64^ Figures displaying alignment of Trinity transcripts to the HPV genome were created using genoPlotR.^68^ Finally, transcripts were translated into all 6 reading frames using the R package seqinr^65^ and appended to the Human UniProt protein reference to create a sample-specific protein database for PEAKS analysis.

GenBank references HPV16 (K02718.1) and HPV18 (X05,015.1) were used for: RNAseq pipeline including Trinity, BLAST alignment of Trinity transcripts, and the HPV genoplot alignment figures with the rainbow of annotated genes at the top. PAVE references were used for: HPV-typing with HPV-EM program, and the associated read counts (in the attached file) and coverage plots.

#### Generation of STCLs and IFNγ ELISpot

PBMCs (1-2×10^6^) were cultured at 37°C and 5% CO2 for 10 days in R10 medium (RPMI with 10% fetal calf serum) plus IL-7 (25 ng/ml) with HPV peptides identified from the cervical cancer panel (4 μg/ml) or a pool of irrelevant FEC peptides (2 μg/ml). Cells were supplemented with 1,800 U/ml IL-2 on days 3 and 7 and additional R10 on day 7. Cells were collected on day 10, washed four times with PBS and rested at 37°C and 5% CO2 for 29–34 h in fresh R10. Elispot plates (MSIPS 4510, Millipore) were coated with anti-human IFN-γ coating antibody (clone 1D1K, MabTech) in the dark at 4°C overnight. Plates were blocked with R10 complete medium at 37°C for a minimum of 2 h. Cells (1 × 10^5^/well) were added and incubated with HPV peptides (2ug/ml) or FEC (4ug/ml). PHA at 5 μg/ml and R10 (0.1% DMSO) were used as positive and negative control, respectively. Plates were incubated for 16–18 h at 37°C, after which a biotinylated anti-human IFN-γ detection antibody (clone 7-B6-1, MabTech) was added for 1 h at room temperature. Plates were then washed 6 times and ABC complex added for 1 h at room temperature followed by the addition of AMEC Complex Buffer. Spot-forming units were counted using an automated reader (AID). The magnitude of response was defined as the number of spot-forming units (SFU) per million PBMCs following background subtraction.

## Data Availability

• The mass spectrometry proteomics data have been deposited to the ProteomeXchange Consortium via the PRIDE (Perez-Riverol et al., 2019) partner repository with the ID PXD028738 and https://doi.org/10.6019/PXD028738. • All custom code for applying existing software packages to our datasets is available upon request to the lead contact.

## Supplemental information

Document S1. Figure S1

Data S1. 4-digit HLA-genotyping of cervical cancer cell line panel

Data S2. 4-digit HLA genotyping of primary cervical cancer patients
