# Supplementary material for: Novel canonical and non-canonical viral antigens extend current targets for immunotherapy of HPV-driven cervical cancer
Source: iScience. 2023 Feb 2;26(3):106101. doi: 10.1016/j.isci.2023.106101 (PMC9978627; doi:10.1016/j.isci.2023.106101)
Supplement: Document S1. Figure S1 [file mmc1.pdf]

iScience, Volume *26*

Supplemental information

Novel canonical and non-canonical viral antigens extend current targets for immunotherapy of HPV-driven cervical cancer

Xu Peng, Isaac Woodhouse, Gemma Hancock, Robert Parker, Kristina Marx, Julius Müller, Silvia Salatino, Thomas Partridge, Annalisa Nicastri, Hanqing Liao, Gary Kruppa, Karin Hellner, Lucy Dorrell, and Nicola Ternette

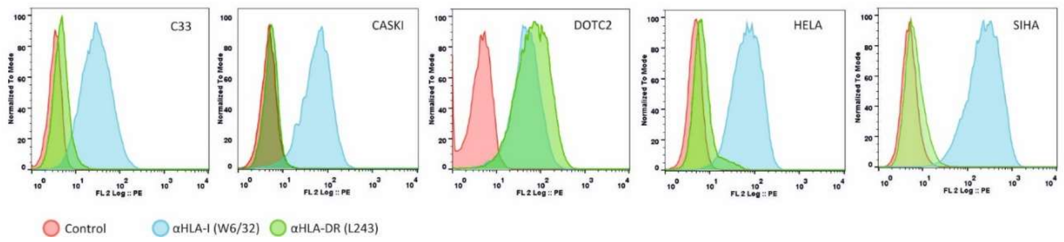

Supplementary Figure S1 (related to Figure 1): Flow cytometry analysis of MHC I and II expression in the cervical cancer cell line panel. *Flow cytometry plots showing the HLA-I (W6/32; blue) and HLA-DR surface expression (L243; green) of the indicated cell lines. Corresponding unstained control cells are shown in red. Plots are gated on live single cells.*
